# The microRNA-124-iGluR2/3 pathway regulates glucagon release from alpha cells

**DOI:** 10.18632/oncotarget.8270

**Published:** 2016-03-22

**Authors:** Haiyang Zhang, Rui Liu, Ting Deng, Xia Wang, Hongmei Lang, Yanjun Qu, Jingjing Duan, Dingzhi Huang, Guoguang Ying, Yi Ba

**Affiliations:** ^1^ Tianjin Medical University Cancer Institute and Hospital, National Clinical Research Center for Cancer, Key Laboratory of Cancer Prevention and Therapy, Tianjin 300060, China; ^2^ Department of Endocrinology, Chengdu Military General Hospital, Chengdu, Sichuan 610083, China

**Keywords:** glucagon, α cell, iGluR2/3, miR-124, metabolism

## Abstract

Glucagon, secreted from islet alpha cells, plays an important role in regulating glucose homeostasis; however, the molecular mechanism underlying this process is not fully understood. Previous studies have demonstrated that miRNAs are involved in the function of alpha cells. Glutamate promotes glucagon secretion by mediating the opening of Ca^2+^ channels. In this present, iGluR2 and iGluR3 levels were significantly increased in fasting-treated mouse islets. Additional studies showed that miR-124-3p simultaneously regulates the expression of iGluR2 and iGluR3 through the direct targeting of mRNA 3’UTR of these two genes. The miR-124-iGluRs pathway also contributed to the high level of glucagon secretion through long-term high glucose levels. Thus, a novel pathway comprising miRNA, glutamate and iGluRs has been demonstrated to regulate the biological process of glucagon release.

## INTRODUCTION

Human blood glucose plays an important role in the control of physiological functions and is regulated through the conversion of pancreatic hormones secretion from different cell types in islets. Insulin and glucagon, secreted from pancreatic beta and alpha cells, respectively, are two important hormones that regulate glucose homeostasis. Generally, a reduction in blood glucose level promotes the release of glucagon from α cells [4, 5], while high blood glucose boosts insulin release from β cells. Previous studies have been focused on the molecular mechanisms underlying insulin synthesis and release [6, 7]; however, the biological process of glucagon secretion is not fully understood.

Recent studies have demonstrated that glutamate secreted from α cells is essential for glucagon release [8]. Glutamate is a major excitatory neurotransmitter in the central nerve system and has been demonstrated as a stimulator of glucagon release in islets [9]. Studies have shown that pancreatic α cells express glutamate, and glutamate is secreted into the vesicles together with glucagon [10, 11]. Glutamate acts on AMPA/kainite type ionotropic glutamate receptors (iGluRs), followed by the opening of Ca^2+^ channels, the increase of the Ca^2+^ concentration in the cytoplasm, and the enhanced secretion of glucagon. Among the ionotropic glutamate receptors, iGluR2 and iGluR3 are located on the α cells of islets [5]. However, the molecular mechanisms that regulate iGluRs expression in the process of glucagon release remain unknown.

In the present study, we examined the expression of iGluR2 and iGluR3 in the islets of mice fasted for 12 h, and both proteins showed a gradual increase within the 12 hours, accompanied by an augment of plasmic glucagon. Isolated mouse islets were treated with 5.6 (low glucose) and 25 mM glucose (high glucose), respectively, and the expression of the two receptors was relatively higher when islets were cultured in low glucose. Consistent with the results obtained from bioinformatics, miR-124-3p is the upstream regulator of both iGluR2 and iGluR3, and the concentration of this molecule was strongly inhibited in the islets of mice after fasting. Subsequent experiments suggested that miR-124-3p simultaneously regulates the expression of iGluR2 and iGluR3 through direct binding with the mRNA 3’UTR, thereby reducing glucagon release. The miR-124-3p-iGluRs pathway was also implicated in enhanced glucagon secretion from islets treated long-term under high glucose, an effect referred to as glucotoxicity, which leads to the dysfunction of both α and β cells in type II diabetes. Therefore, the results of the present study provided information concerning the network that regulates glucagon release, and miR-124-3p is a potential therapeutic drug target for the reduction of glucagon in diabetes mellitus.

## RESULTS

### Low blood glucose promotes the expression of iGluRs

**Figure 1:**
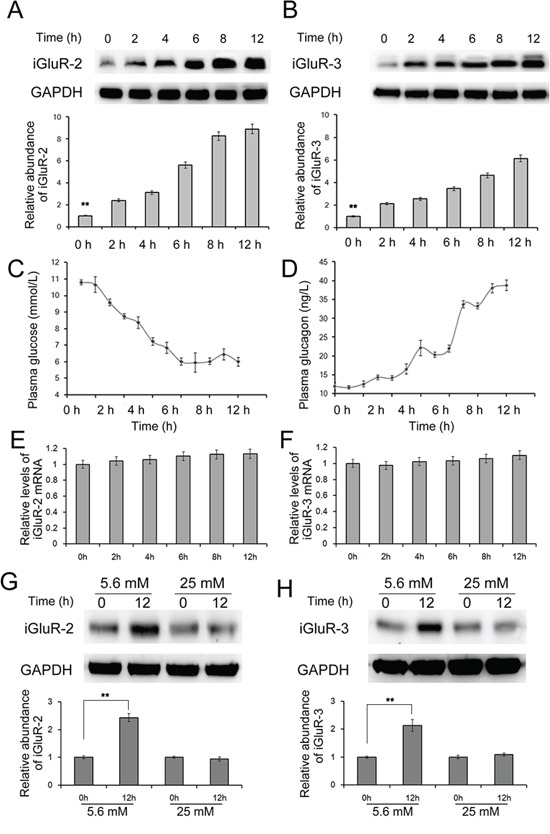
The effects of decreased glucose on the expression of iGluR2/3 20 C57/BL6 mice were fasted for 12 hours, and islets were isolated to determine expression of iGluR2 **A.** and iGluR3 **B.** through western blotting (n=5); **C-D.** the blood glucose (C) and blood glucagon (D) levels were also detected (n=5); **E-F.** relative mRNA levels of iGluR2 (E) and iGluR3 (F) in mouse islets (n=5). **G-H.** Isolated mouse islets were cultured in DMEM containing different concentrations of glucose (n=5). The protein levels of iGluR2 (G) and iGluR3 (H) in mouse islets treated with 5.6/25 mM glucose. ** indicates p<0.01.

Studies have reported that iGluR2 and iGluR3 are expressed in pancreatic α cells but not in beta cells, and these receptors positively regulate glucagon release14. To evaluate the expression of iGluR2 and iGluR3 in alpha cells when blood glucose levels are reduced, the mice were fasted for 12 h, and the islets were isolated, followed by western blot analysis. As shown in Figure 1A and B, the expression of both iGluR2 and iGluR3 displayed a significant change: iGluR2 increased 7-fold and iGluR3 increased nearly 6-fold within 12 hours.

We also detected the mRNA levels of the iGluR2 and iGluR3 and observed that iGluR2 mRNA remained unchanged (Figure 1E and 1F). These data suggest that blood glucose is a regulator of iGluR2 and iGluR3 in α cells; a reduction in the blood glucose level increased the expression but did not affect the transcription of these two proteins.

### The effects of fasting on plasma glucose and glucagon

Fasting decreases plasma glucose and induces glucagon secretion from pancreatic α cells. In the present study, the glucose and glucagon levels in the blood were measured every hour. As expected, the blood glucose level was gradually decreased, while the blood glucagon level showed a sensitive increase during fasting. The blood glucose level decreased from 10 mmol/L at 2 h to 6 mmol/L at 9 h, which was slightly reversed within the following 3 h (Figure 1C). The blood glucagon level increased from 40 to 70 ng/L, accompanied with a change in glucose levels (Figure 1D). This result was consistent with previous studies [12, 13], confirming that fasting decreases blood glucose and subsequently regulates the expression of iGluR2 and iGluR3.

### High glucose suppresses iGluR2 and iGluR3 expression in isolated islets

To obtain a direct understanding of the glucose-regulating-iGluRs expression in α cells, we also treated isolated islets with high glucose (25 mM) and low glucose (5.6 mM) levels. Islets were cultured in DMEM containing the desired amounts of glucose, followed by harvesting at 12 h. The protein level of iGluR2 was increased in islets treated with low glucose levels, while only a slight effect was observed in islets treated with high glucose levels (Figure 1G); similar observations were obtained for iGluR3, which showed a mild decrease in 25 mM glucose-treated islets (Figure 1H). This observation suggests that low glucose induces while high glucose inhibits iGluR expression in pancreatic α cells.

### The prediction of iGluR2/3-related miRNAs

**Figure 2:**
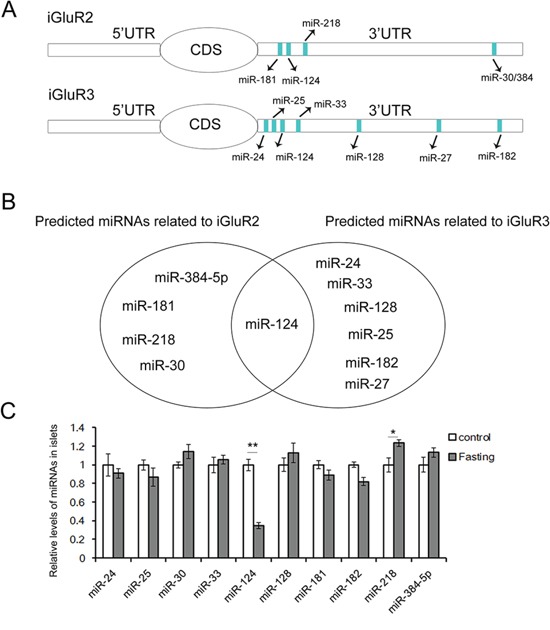
The predicted upstream miRNAs associated with iGluR2/3 The upstream miRNAs of iGluR2 and iGluR3 are predicted using Targetscan, and the expression of these miRNAs in islets of fasted mice is determined by qRT-PCR. **A.** The miRNAs predicted as potential regulators of iGluR2 and iGluR3. **B.** miR-124-3p is the common regulator of iGluR2 and iGluR3. **C.** Relative levels of iGluR2/3-related miRNAs (n=5). ** indicates p<0.01; * indicates p<0.05.

It is clear that rapid glucagon and insulin secretion is primarily regulated through autocrine factors, and endogenic miRNAs are involved in insulin synthesis and release [6, 10]. Therefore, the aim of the present study was to identify the potential miRNAs that regulate glucagon secretion. The predicted upstream miRNAs of iGluR2/3 are listed in Figure 2A, and miR-124-3p was shared between both iGluR2 and iGluR3 (Figure 2B).

The levels of these miRNAs in islets of fasted mice are detected by qRT-PCR, and it is showed that miR-124 expression is suppressed (Figure 2C).

### GluR2 and iGluR3 are direct targets of miR-124-3p

**Figure 3:**
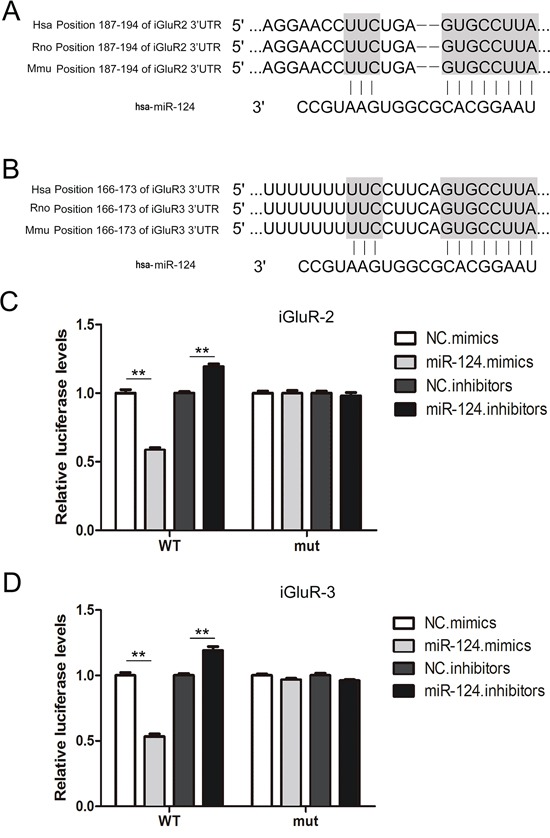
Identification of iGluR2/3 as direct targets of miR-124-3p using luciferase assays **A-B.** Schematic description of the hypothetical duplexes formed by interactions between miR-124-3p and the 3’UTR of iGluR2 (A) and iGluR3 (B). **C-D.** Direct recognition of iGluR2/3 through miR-124-3p (n=5). Firefly luciferase reporters containing either WT or mutant iGluR2 (C) and iGluR3 (D) 3’UTR sequences were co-transfected into HEK293T cells with scrambled non-coding RNA (NC) miRNA mimics, or miRNA inhibitors. At 24 h post-transfection, the cells were assayed using luciferase assay kits. ** indicates p<0.01.

To provide direct evidence of the interaction between miR-124-3p and iGluR2/3, a luciferase assay were performed to evaluate this association. The predicted binding sites in the 3’UTR of iGluR2 and iGluR3 were highly conserved among humans, mice and rats (Figure 3A and 3B). Co-transfection with miR-124-3p mimics the reporter plasmid containing WT iGluR2 3’UTR in cells, resulting in a nearly 50% decrease in luciferase activity, and miR-124-3p mimics also reduced the luciferase signal by 40% when co-transfected with iGluR3 3’UTR-containing plasmids; the luciferase signal showed a slight increase when miR-124-3p expression was down-regulated (Figure 3C and 3D). However, the inhibition of luciferase activity was lost when the binding sites were mutated (Figure 3C and 3D). These data showed that miR-124-3p simultaneously targets the 3’UTR of iGluR2 and iGluR3.

### miR-124-3p regulates glucagon release though the repression of iGluR2/3 expression

**Figure 4:**
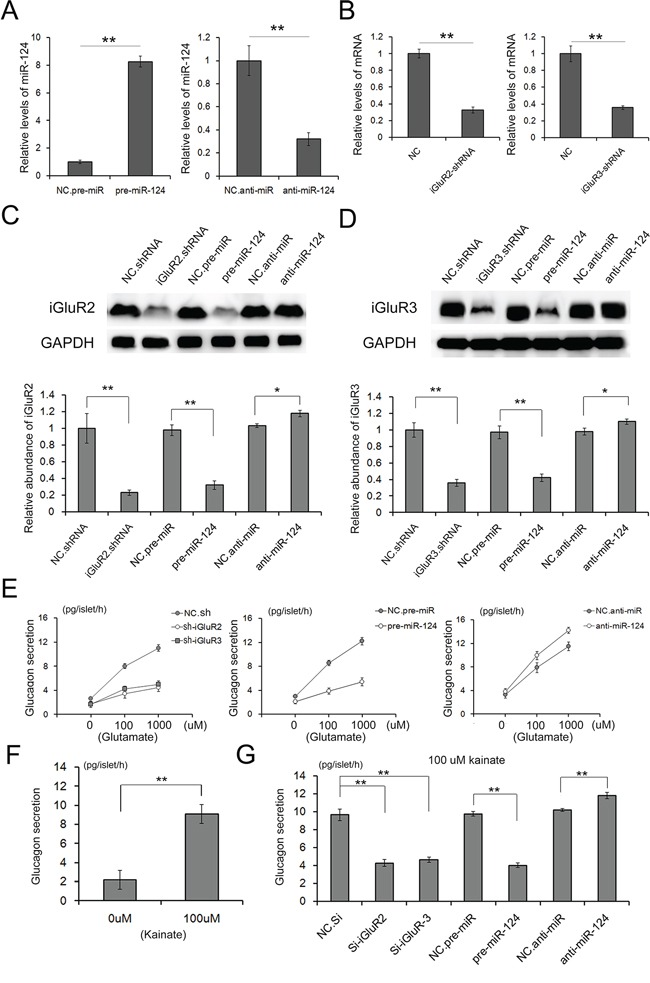
Role of miR-124-3p in glucagon release through iGluR2/3 targeting Lenti-virus particles are used to overexpress or inhibit miR-124 expression in islets; and lenti-virus particles containing shRNA are used to suppress the expression of iGluR2/3 expression. **A.** relative levels of miR-124 in islets treated with pre- or anti-miR-124 (n=3). **B.** Relative levels of iGluR2/3 mRNA in islets treated with shRNAs (n=3). **C** and **D.** The expression of iGluR2 (C) and iGluR3 (D) in isolated islets transfected with shRNA or pre/anti-miR-124-3p mediated through lentiviral particles (n=5). **E-G.** Glutamate and kainite-stimulated glucagon release from islets transfected with iGluR2/3 shRNAs or pre/anti-miR-124-3p (n=5). ** indicates p<0.01.

Mammalian miRNAs inhibit protein expression at the post-transcriptional level [14, 15]. We used a lentivirus containing a pre/anti-miRNA sequence to suppress the miR-124-3p level in islets. The expression of iGluR2/3 was assessed using western blot analysis at 24 h post-transcription. As is shown in Figure 4C and 4D, the overexpression of miR-124-3p apparently suppressed iGluR2/3 levels, and iGluR2 and iGluR3 were reduced by 65% and 60%, respectively; the inhibition of miR-124-3p showed only a slight increase in the expression of the two proteins. siRNA was also used as a positive control to abate protein expression, and the expression of both proteins decreased 30%.

Glutamate and kainate are two stimulators of α cell function, which significantly improve glucagon secretion via iGluRs [8, 16]. Glutamate stimulated high, concentration-dependent increases in glucagon release, showing 8 pg/islet/h at 100 μM glutamate and 12 pg/islet/h at 1000 μM glutamate (Figure 4E). However, the glutamate-sensitive glucagon release was blocked when iGluR2 and iGluR3 were strongly inhibited through either siRNA or miR-124-3p (Figure 4E). Increases in glucagon release could also be elicited through 100 μM kainite (Figure 4F), and the kainite-stimulated glucagon release was also eliminated through the inhibition of iGluRs expression (Figure 4G).

Both glutamate and kainite were demonstrated as effective stimulators of α cell function, and miR-124-3p largely reduced glucagon secretion levels via the synchronous suppression of iGluR2 and iGluR3 expression.

### The effects of glucotoxicity on miR-124-3p and iGluR2/3

**Figure 5:**
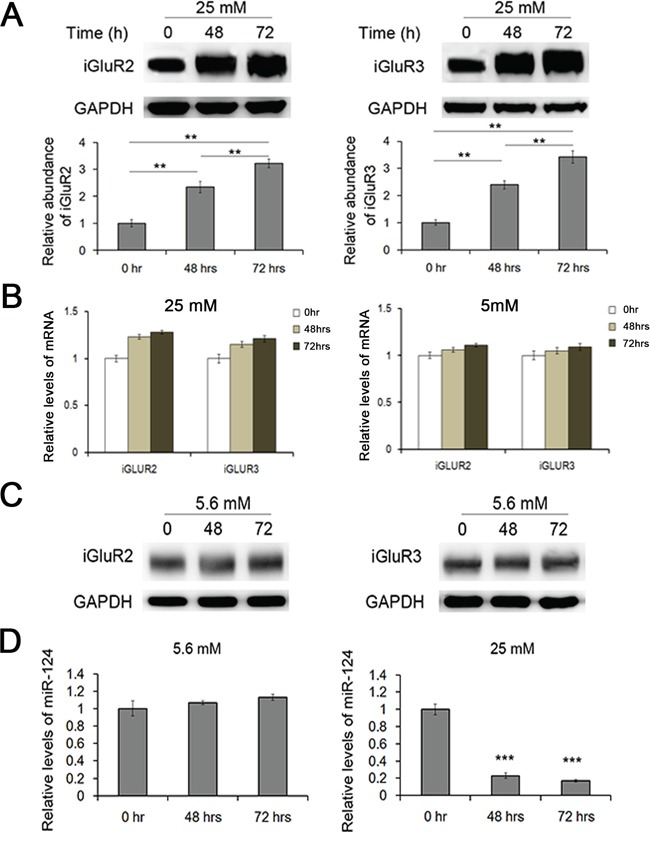
Effects of glucotoxicity on the expression of miR-124-3p and iGluR2/3 Mouse islets were cultured in medium containing 25 mM and 5.6 mM glucose for 48/72 hrs. **A.** The expression of iGuR2/3 in islets treated with long-term high glucose (n=5). **B.** Relative levels of iGluR2/3 mRNA in islets cultured in long-term glucose (25 mM and 5.6 mM) (n=5). **C.** The expression of iGluR2/3 protein in islets treated with 5.6 mM glucose for 48/72 hrs (n=5). **D.** The expression of miR-124-3p in islets cultured under long-term high glucose (n=5). ** indicates p<0.01; *** indicates p<0.001.

Type II diabetes is characterized through decreased insulin secretion, long-term high blood glucose and increased glucagon release [17, 18]. Long-term exposure to high glucose, known as glucotoxicity, typically leads to the dysfunction of a wide range of organs, including pancreatic α and β cells [19–22]. In previous studies, miRNAs have been implicated in decreased insulin secretion resulting from pro-longed high glucose [14]. In the present study, mouse islets were cultured in medium containing 25 and 5.6 mM glucose for 72 h, and the expression of both iGluR2/3 and miR-124-3p was detected. Both proteins increased two-fold at 48 h and nearly three-fold at 72 h (Figure 5A) but remained unchanged in 5.6 mM glucose (Figure 5C), while miR-124-3p was strongly inhibited under prolonged high glucose (Figure 5D). As is respected, mRNA levels of iGluR2 and iGluR3 showed little change under the treatment of long-term glucose (Figure 5C). This observation suggested that the miR-124-3p-iGluR pathway also contributed to the high glucagon secretion in type II diabetes.

## DISCUSSION

In recent years, miRNA associated pathways have been largely explored in various biological processes. Recent studies have shown that miRNAs are also involved in pancreatic β cell function [23–25], but the role of miRNAs in α cells has not yet been reported. Generally, the change in blood glucose results in the release of insulin or glucagon, which have opposite functions in regulating glucose homeostasis. However, the regulation of glucagon secretion is far more complex than a single glucose switch. Glutamate has been established as a bona fide autocrine signaling molecule in α cells, providing positive feedback for glucagon release from islets [26–28], which stimulates glucagon-like peptide 1 secretion and reduces postprandial glucose [29–31].

The glutamate-iGluRs signaling pathway explains the modest decrease in plasma glucose, which effectively increases glucagon secretion; however, the upstream regulation of iGluRs has not yet been explored. The results of the present study demonstrated that a decrease in blood glucose induces the expression of iGluR2/3. The expression of iGluR2/3 increased, accompanied by a sustained loss of blood glucose, and the mRNA levels remained unchanged. Islets cultured in high glucose also showed lower iGluRs expression, and miR-124-3p was a common regulator of both iGluR2 and iGluR3, which are specifically distributed in α cells but not in β cells.

We used lentiviral particles to mediate the transfection of shRNA and pre/anti-miRNA into isolated islets, and this method was effective. The disrupted expression of iGluR2/3 through shRNA resulted in a sharp decrease in glucagon secretion from islets and reduced the sensitivity to glutamate concentration, indicating that both iGluR2 and iGluR3 are necessary and ireplaceable during glucagon release. The overexpression of miR-124-3p also decreased iGluR2/3, effectively suppressing the secretion of glucagon. The luciferase assays confirmed that miR-124-3p directly targets the 3’UTR of iGluR2/3. Therefore, miR-124-3p is involved in α cell function through the simultaneous regulation of iGluR2 and iGluR3 expression.

Prolonged high glucose is referred to as glucotoxicity, a condition that decreases insulin secretion from islets [32–34]. In the present study, we confirmed that glucotoxicity enhanced glucagon secretion from islet α cells, with increased expression of iGluR2/3 and down-regulation of miR-124-3p. The persistent high glucose of type II diabetes leads to the dysfunction of organs and tissues, including β and α cells. Thus, it has been suggested that the pathway comprising miR-124-3p and iGluR2/3 contributes to the functional disorder of α cells in type II diabetes.

Herein, we demonstrated that miR-124-3p is the common upstream regulator of iGluR2 and iGluR3, thus governing the physiological process of glucagon secretion. The miR-124-3p-iGluR2/3 pathway might also be involved in α cell dysfunction. This model explains, at least in part, how endogenic miRNAs effectively regulate glucagon release from human pancreatic α cells. Future studies are needed to uncover more miRNA-related targets and enhance the current understanding of the molecular mechanism of α cell function.

## MATERIALS AND METHODS

### Animals

C57BL/6J mice were housed in a pathogen-free animal facility with controlled temperature (22 ± 1¼C) and lighting (lights on 6:00 AM to 6:00 PM). All of the experimental procedures were performed in accordance with protocols approved through the Institutional Animal Care and Research Advisory Committee of the Third Military Medical University.

### Determination of plasma glucose

Blood glucose was assayed in tail blood using a free-style glucometer (Therasense, Alameda, CA, USA).

### Islet isolation and culture

Mouse islets were isolated as previously described, with slight improvements [35, 36]. 20 mice were anesthetized through the intraperitoneal injection of sodium pentobarbital. Pancreatic islets were subsequently isolated through pancreatic duct injection of 500 U/ml of collagenase solution, followed by digestion at 37¼C for 28 min with mild shaking. The islets were washed several times with D-Hanks (136 mM NaCl, 0.53 mM KCl, 4.2 mM NaHCO_3_, 0.44 mM KH_2_PO_4_, and 0.385 mM Na_2_HPO_4_), separated from acinar cells on a discontinuous Ficoll 400 gradient, and viewed under a dissecting microscope, followed by hand selection. Moreover, the islets were collected and transferred into RPMI 1640 medium containing 10% fetal bovine serum.

The isolated islets were cultured at 37¼C in a humidified atmosphere containing 5% CO_2_/95% air for 12 h (primary culture) to remove exocrine and other tissues. Subsequently, the islets were transferred to Dulbecco's modified Eagle's medium (DMEM) containing 5.6/25 mM glucose and 10% FBS.

### Islet transfection with lenti-virus

Lentiviral particles containing the shRNAs of iGluR2 and iGluR3 were obtained from Santa Cruz (sc-35488-V and sc-35490-V); lentiviral particles containing pre-miR-124-3p and anti-miR-124-3p were synthesized at GenePharm (Shanghai). A total of 100 mouse islets were cultured in medium as described above, and subsequently 10^7^ lentiviral particles were added to the medium with gentle mixing. The total protein or RNA was isolated from islets after 48 hours.

### ELISA assay for the determination of glucagon

The concentration of glucagon in the mouse serum and medium were assessed using the Glucagon EIA Kit (Sigma, RAB0202-1KT). Briefly, the samples were added to a 96-well plate coated at the bottom with glucagon antibody. A total of 20 μl of assay buffer, 10 μl of glucagon standards/medium, and 80 μl of detection antibody were added to the samples. Subsequently, 100 μl of enzyme buffer and 100 μl of substrate solution buffer were added to each well. After a final stop solution was added, the glucagon content was determined based on the absorbance measured at 450 nm.

### RNA isolation and quantitative RT-PCR

Assays to quantify mature miRNAs were conducted as previously described [37]. Total RNA was extracted from the cultured cells using TRIzol Reagent (Invitrogen) according to the manufacturer's instructions.

In the present study, miRNA expression in the cells and islets was normalized to that of U6 snRNA [38]; and mRNA expression was normalized to GAPDH. The relative amount of each gene to the internal control was calculated using the equation 2^−ΔCT^, in which ΔCT=C_T gene_-C_T control_. Real-time PCR was performed using Taqman probes (miRNA) and subgreen (mRNA) on a 7500 or 7300 PCR system (Applied Biosystems). The cut-off value for both miRNA and mRNA is 35.

### Plasmid construction and luciferase assay

A segment of the iGluR2 and iGluR3 3’-untranslated region (UTR) was synthesized and inserted into the p-MIR-report plasmid (Ambion), and the plasmid with a mutated 3’UTR was produced using the same method. For luciferase reporter assays, 2 μg of firefly luciferase reporter plasmid, 2 μg of beta-galactosidase expression vector (Ambion), and equal amounts (200 pmol) of mimics, inhibitors, or scrambled negative control RNA were transfected into the cells in 6-well plates. The beta-galactosidase vector was used as a transfection control. At 18 h after transfection, the cells were assayed using luciferase assay kits (Promega).

### Western blotting

The expression of iGluRs was assessed through western blot analysis, and the samples were normalized to GAPDH. Protein extraction was blocked using PBS-5% fat-free dried milk at room temperature for 1 h and incubated at 4¼C overnight with anti-iGluR2 (1:1000, Santa Cruz), anti-iGluR3 (1:1000, Santa Cruz), anti-GAPDH (1:2000, Santa Cruz) antibodies, respectively.

### Target prediction

TargetScan (http://www.targetscan.org) was used to predict the biological targets of miRNAs based on the presence of conserved 8-mer and 7-mer sites matching the seed region of each miRNA.

### Statistical analysis

All data were representative of at least three independent experiments. The data were expressed as the means ± SD of three separate experiments. p < 0.05 was considered statistically significant using Student's *t*-test. ** indicates p < 0.01; *** indicates p < 0.001.
